# Successful enteral nutrition in the treatment of esophagojejunal fistula after total gastrectomy in gastric cancer patients

**DOI:** 10.1186/1477-7819-8-71

**Published:** 2010-08-16

**Authors:** Michel Portanova

**Affiliations:** 1 Gastric Cancer Service, Department of General Surgery, Rebagliati National Hospital, Lima, Perú

## Abstract

**Background:**

Esophagojejunal fistula is a serious complication after total gastrectomy in gastric cancer patients. This study describes the successful conservative management in 3 gastric cancer patients with esophagojejunal fistula after total gastrectomy using total enteral nutrition.

**Methods:**

Between January 2004 to December 2008, 588 consecutive patients with a proven diagnosis of gastric cancer were taken to the operation room to try a curative treatment. Of these, 173 underwent total gastrectomy, 9 of them had esophagojejunal fistula (5.2%). In three selected patients a trans-anastomotic naso-enteral feeding tube was placed under fluoroscopic vision when the fistula was clinically detected and a complete polymeric enteral formula was used.

**Results:**

The complete closing of the esophagojejunal fistula was obtained in day 8, 14 and 25 respectively.

**Conclusion:**

In some selected cases it is possible to make a successful enteral nutrition using a feeding tube distal to the leak area inserted with the help of fluoroscopic vision. The specialized management of a gastric surgery unit and nutritional therapy unit are highlighted.

## Background

The dehiscence of an esophagojejunal anastomosis is one of the major complications after a total gastrectomy in gastric cancer. It is associated with high mortality. When referring to esophagojejunal anastomosis, most studies approach the procedure. That is, if to use hand-sutured or mechanical-stapled, and how this can influence the origin of an anastomotic dehiscence, and also explores the risk factors for the presentation of this complication [1,2]. Nevertheless there are practically, no reports of the management of such complication. When this complication arises the few reports that exist agree that the use of parental nutrition is the first option. In our understanding, this is the first complete report of successful enteral nutrition for the treatment of a fistula of the esophagojejunal anastomosis following total gastrectomy in gastric cancer.

## Methods

From January 2004 to December 2008, 588 patients with confirmed diagnosis of gastric cancer were taken to the operation room to try a curative treatment in the Gastric Cancer Service of the National Rebagliati Hospital in Lima, Peru. Of these, 173 underwent total gastrectomy, 9 of them had esophagojejunal fistula (5.2%)

The diagnosis of this complication was suspected because of the characteristics of the discharge obtained from the drain that was inserted during the intraoperative act and proved by administering 20 cc of water with methylene blue, and observing the immediate exit of it through the drain, located inside the abdomen.

Six patients were in poor general status with signs of sepsis; even some of them must be transferred to the intensive care unit or taken to operating room. None of them was chosen for this study.

**Figure 1:**
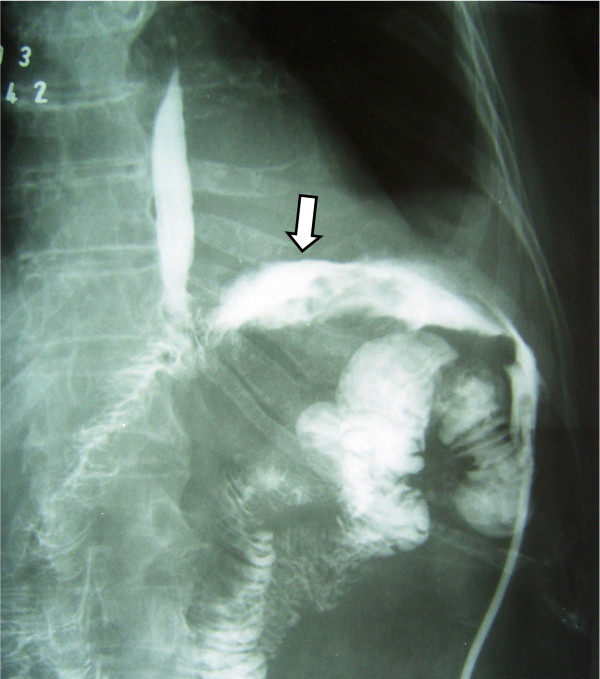
**The esophagus and jejunum**. The arrow indicates the fistula duct.

**Figure 2:**
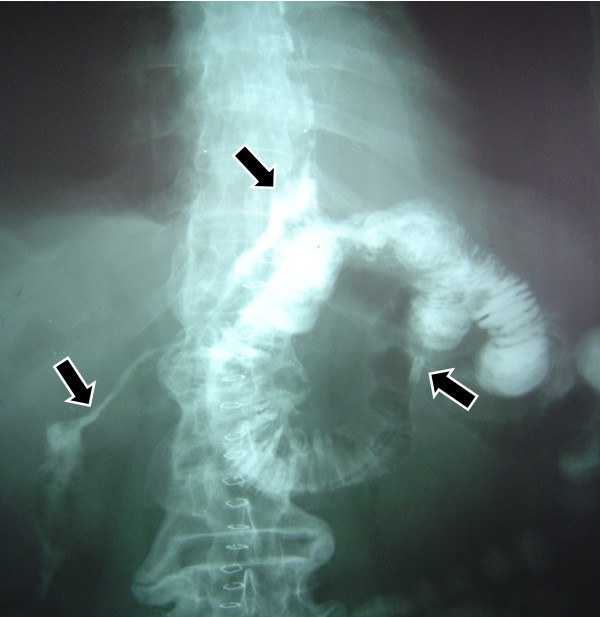
**The leak area, fistula duct and the distal portion of the feeding tube located in the jejunum away from the leak area**. Each point is indicated by arrows.

Three patients in good general condition and without signs of sepsis, regardless output volume of the fistula, were taken to the X ray room, and under fluoroscopy were administered contrast substance through oral route to reconfirm and document the diagnosis of the fistula (Figure 1), immediately after this, a naso-enteral feeding tube French 10 was inserted and located distally to the dehiscence of the anastomosis, always under fluoroscopy. In Figure 2 you can appreciate the leaking area, the fistula duct, as well as the naso-enteral tube located distally to the leaking area. It was verified that the tube was in the intestinal lumen, by injecting hydro soluble contrast substance to visualize the jejunal mucosa. Through the feeding tube, a complete polymeric isotonic enteral nutrient was administered, in a dose of 1.5 grams of protein per kilogram of weight per day, by using an infusion bomb during 20 hours, (with a resting time of 4 hours).

## Results

The complete closing of the fistula was evident in day 8, 14 and 25 respectively, when no discharge was detected from the drain. No complications were detected during the treatment period. The patients could start their oral feeding without any inconvenience and were discharged in good conditions afterwards.

## Discussion

Gastric cancer is a very common cancer worldwide and surgery is the only treatment modality offering hope for cure. Sometimes, because of the location and characteristics of the tumor, such surgical treatment implies a total gastrectomy associated with an excision of the regional lymph nodes. It is a surgery of a high level of complexity that has a risk of death or complication and the dehiscence and fistula of the esophagojejunal anastomosis is one of the most feared complications. Reports show that this complication presents in 7 to 15% of the operated patients [3-5].

Although randomized studies do not show a difference in the presentation of this complication in this anastomosis where as if done manually or using stapler devices [6], a recent analysis of a great series of the National Cancer Center of Tokyo, emphasized that there is a learning curve in the using of an automatic suture and once this phase is surpassed, the presentation of this complication is between 0-1% suggesting that currently the use of stapler for this anastomosis has to be considered the gold standard [7].

Stapler devices are routinely used in our Hospital, but sometimes we don't have these devices available. In these cases a hand sewn anastomosis is performed. Within the three patients in the study group, two of them had a hand-sewn anastomosis.

The dehiscence of the esophagojejunal anastomosis is associated with a high mortality that can even get to 30% [8]. A series of reports show the importance of the specialization and experience of the surgical group, not only to lower the morbidity and mortality of the gastrectomy but also because of the complications that can arise and have to be approached in a successful way [9]. In the most complicated cases, more aggressive measures should be taken, like intensive therapy, re-laparotomy, administration of antibiotics of most recent generation, etc. In less serious cases, it is important to have from the beginning a conservative management, establishing the basic support measures that include absolute restriction of oral route and the administration of antibiotics.

From the point of view of the nutritional assistance that these patients require, it always has been understood that we should recur immediately to total parenteral nutrition, and that enteral nutrition is a contraindication in this case. The scarce tendency towards the use of enteral nutrition in these cases is not because enteral nutrition per se, but basically because of the fear of putting a tube that has to cross the dehiscence area to make this therapeutic alternative possible. This is the cause that many surgeons choose to leave routinely a transanastomotic tube during the intraoperative time [10], although a recent meta-analysis shows that the rutinary use of a tube in the gastrectomy surgery is not justified [11]. In the other hand, the ileus that many times is associated with this complication and the subsequent reflux of the nutrient to the fistula opening is limiting for the use of enteral therapy, even if the tube is located away from the dehiscence area.

It is important to highlight that the National Hospital Rebagliati of Lima, Peru, where this investigation took place, part of the surgical team of the Gastric Cancer Service is also integrated to the Specialized Unit of Artificial Nutrition Therapy and thus has a great experience in nutritional assistance techniques as the application of naso-enteral tubes using fluoroscopic guide. This is the reason that in these three cases, it was decided to try enteral nutrition, which was initiated after putting the feeding tube. On the other hand, fluoroscopically guided percutaneous jejunostomy could be a good alternative in these cases, but has never been performed at our hospital.

**Figure 3:**
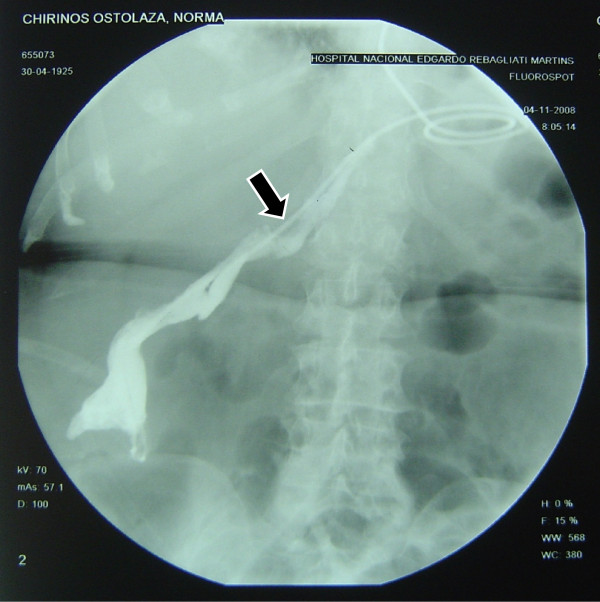
**The feeding tube in the wrong location into the fistula duct**.

**Figure 4:**
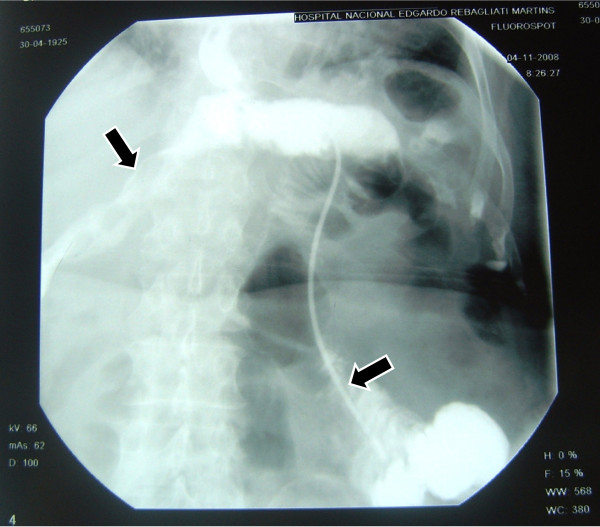
**The fistula duct and the same feeding tube correctly placed into the jejunum**.

**Figure 5:**
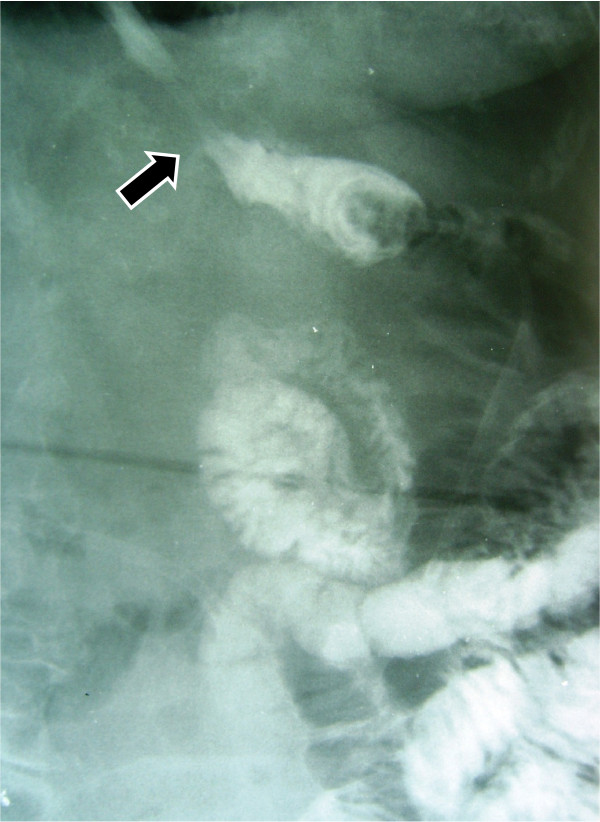
**Complete closing of the fistula**. No leak is detected.

In one of our cases, when putting the tube in place, this initially exited through a dehiscent area and crossed the route of the intraabdominal fistula (Figure 3), but it was re oriented to the adequate place, in the intestinal lumen, distal to the zone of escape (Figure 4). This emphasizes the importance of experience in the handling of this alternative therapy. In this case, a radiographic control can be seen after the successful closing of the escape (Figure 5).

The nutrient that was used was a complete liquid polymeric enteral formula, in a dose of 1.5 grams of protein per kilo of weight per day. An infusion bomb was used to deliver the formula.

## Conclusions

The dehiscence of the esophagojejunal anastomosis post total gastrectomy is a serious complication associated with a high mortality. In some selected cases it is possible to make a successful enteral nutrition using a feeding tube distal to the leak area inserted with the help of fluoroscopic vision. These patients should be managed with an expert multidisciplinary team.

## Competing interests

The author has no commercial or financial interest or financial conflict with the subject matter or materials discussed in this manuscript.
